# Error in Subtitle

**DOI:** 10.1001/jamahealthforum.2021.2479

**Published:** 2021-08-13

In the Viewpoint titled “Balancing Quality and Speed in the Market Approval of Diagnostic Tests,”^1^ published online on July 9, 2021, the subtitle incorrectly listed the countries from which the experiences were drawn. This article was corrected online.
